# Classical ROS-dependent and early/rapid ROS-independent release of Neutrophil Extracellular Traps triggered by *Leishmania* parasites

**DOI:** 10.1038/srep18302

**Published:** 2015-12-17

**Authors:** Natalia C. Rochael, Anderson B. Guimarães-Costa, Michelle T. C. Nascimento, Thiago S. DeSouza-Vieira, Matheus P. Oliveira, Luiz F. Garcia e Souza, Marcus F. Oliveira, Elvira M. Saraiva

**Affiliations:** 1 Laboratório de Imunobiologia das Leishmanioses, Departamento de Imunologia, Instituto de Microbiologia Paulo de Góes, Universidade Federal do Rio de Janeiro, RJ, 21941-902. Brazil; 2 Laboratório de Bioquímica de Resposta ao Estresse, Instituto de Bioquímica Médica, Programa de Biologia Molecular e Biotecnologia, Universidade Federal do Rio de Janeiro, Cidade Universitária, 21941-902, Rio de Janeiro, Brazil; 3 Laboratório de Inflamação e Metabolismo, Instituto Nacional de Ciência e Tecnologia de Biologia Estrutural e Bioimagem (INBEB), Universidade Federal do Rio de Janeiro, RJ, 21941-902. Brazil; 4 Vector Molecular Biology Unit, Laboratory of Malaria and Vector Research, National Institute of Allergy and Infectious Diseases, National Institutes of Health, Rockville, MD, 20852, USA

## Abstract

Neutrophil extracellular traps (NETs) extruded from neutrophils upon activation are composed of chromatin associated with cytosolic and granular proteins, which ensnare and kill microorganisms. This microbicidal mechanism named classical netosis has been shown to dependent on reactive oxygen species (ROS) generation by NADPH oxidase and also chromatin decondensation dependent upon the enzymes (PAD4), neutrophil elastase (NE) and myeloperoxidase (MPO). NET release also occurs through an early/rapid ROS-independent mechanism, named early/rapid vital netosis. Here we analyze the role of ROS, NE, MPO and PAD4 in the netosis stimulated by *Leishmania amazonensis* promastigotes in human neutrophils. We demonstrate that promastigotes induce a classical netosis, dependent on the cellular redox imbalance, as well as by a chloroamidine sensitive and elastase activity mechanism. Additionally, *Leishmania* also induces the early/rapid NET release occurring only 10 minutes after neutrophil-parasite interaction. We demonstrate here, that this early/rapid mechanism is dependent on elastase activity, but independent of ROS generation and chloroamidine. A better understanding of both mechanisms of NET release, and the NETs effects on the host immune system modulation, could support the development of new potential therapeutic strategies for leishmaniasis.

Neutrophils are the most abundant leukocytes in blood and play an important role in the innate immune response. They are the first cells to arrive at an infection site and are endowed with potent antimicrobial mechanisms. Netosis is one of these mechanisms and occurs with the release of a scaffold of chromatin associated with different granular and intracellular proteins, named neutrophil extracellular traps (NETs)^1^,^2^. NET release can be triggered by several stimuli, among them, pathogens such as *Leishmania*^3^,^4^.

The molecular mechanisms behind NET formation are still poorly understood. To date two main NET release mechanisms have been proposed: the classical, reactive oxygen species (ROS)-dependent and the early/rapid ROS-independent^5^,^6^. In the classical mechanism, neutrophils enter a cell death program that culminates with the release of NETs 1–4 hours after activation, in a process that is dependent on ROS production. The majority of the stimuli described to induce netosis are dependent on ROS generation by the NADPH oxidase complex, in such a way that enzymatic inhibitors of this complex abrogate NET release^2^,^7^,^8^,^9^. Furthermore, neutrophils from chronic granulomatous disease patients are unable to release NETs, unless a ROS source is provided^2^,^10^. However, it has been demonstrated that neutrophils can release NETs without the activation of the NADPH oxidase complex. During the early/rapid vital netosis mechanism, neutrophils extrude NETs after as little as 5–15 min of activation, without affecting neutrophil viability^5^. Chromatin decondensation is an essential step towards netosis. Peptidyl arginine deiminase 4 (PAD4), as well as elastase and myeloperoxidase seem to be critical for NET release through the classical pathway^11^,^12^,^13^,^14^. PAD4 catalyzes histone citrullination allowing chromatin decondensation. Therefore, PAD4 knockout (KO) mice are unable to produce NETs^13^,^14^,^15^. Chromatin decondensation may also be accomplished by elastase and myeloperoxidase. After netosis activation, elastase migrates to the nucleus, to digest histones, a process that is further enhanced by MPO^11^. Therefore, elastase KO mice are netosis incapable^11^, and MPO deficient patients have impairment in NET release^12^.

We previously described that *Leishmania amazonensis* promastigotes induce NET release from human neutrophils, are trapped by these scaffolds and killed by the histones associated to these structures^3^. Here we characterize the mechanisms behind NET induction by this parasite. We investigated the participation of elastase, myeloperoxidase and PAD4 on NET formation induced in human neutrophils by *L. amazonensis* promastigotes. ROS involvement in NET induction was analyzed by using inhibitors of ROS/RNS (reactive nitrogen species) producing mechanisms, such as mitochondrial electron transport system, nitric oxide synthase (NOS) and xanthine oxidase. As a control, we have utilized phorbol 12-myristate 13-acetate (PMA), since it was one of the first stimuli described to induce netosis^1^, and a well-known cellular ROS inducer mediated by NADPH oxidase^16^.

Our results demonstrate that *Leishmania* promastigotes trigger the classical netosis, by promoting redox imbalance, with the involvement of NADPH-oxidase and NOS derived ROS/RNS, respectively. This mechanism is also dependent on PAD4 and elastase activity. Furthermore, promastigotes promoted the early/rapid, ROS-independent NET formation occurring only 10 minutes after neutrophil-parasite interaction, which is dependent of elastase, but not on PAD4.

## Results

### Elastase and PAD4 are involved in classical netosis induced by Leishmania

**Figure 1:**
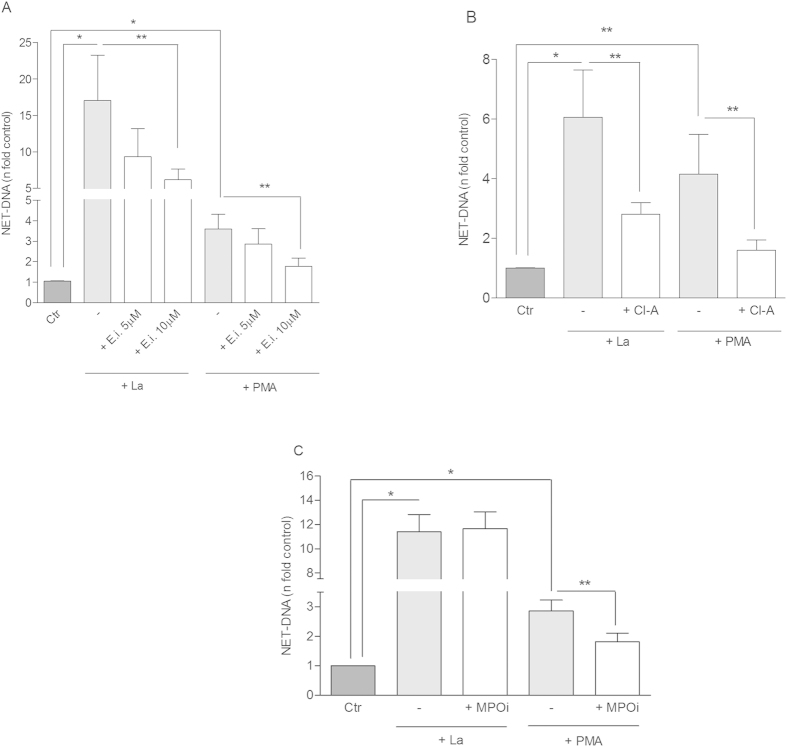
Chloroamidine and elastase inhibitor decreased netosis induced by *Leishmania amazonensis* (La). Neutrophils (NØ; 2 × 10^6^) were incubated with (**A**) Elastase inhibitor (E.i, 5 and 10 μM); (**B**) Chloroamidine (Cl-A, 12 μM) and (**C**) Myeloperoxidase inhibitor (MPOi, 300 nM), for 30 min and then, stimulated or not with PMA (100 nM) or promastigotes of *L. amazonensis* (1NØ: 0.1 La ratio) for 1 h. Following stimulation, DNA quantification was performed using PicoGreen assay kit in culture supernatants. Data normalized regarding spontaneous release of DNA representing the mean ± SEM from 17 (**A**), 7 (**B**,**C**) donors. *p < 0.0001 and **p < 0.05.

After demonstrating that *Leishmania* promastigotes induce NET release by human neutrophils^3^, we were interested to further elucidate the mechanisms involved in this process. Thus, we first assessed the role of elastase, myeloperoxidase and PAD4 on NET induction by *Leishmania*, by pre-treating neutrophils with their respective inhibitors. The cell permeable inhibitor of human elastase, MeOSuAAPV-CMK^17^, decreased netosis induction by *Leishmania* (Fig. 1A). A reduction of 45% and 64% was obtained upon 5 and 10 μM pretreatment with the elastase inhibitor, respectively. Similarly, elastase inhibition decreased 54% netosis induction by PMA (Fig. 1A). Due to the variability in the human donors’ response all results were presented as n fold control, but we also show the donor-to-donor variation as the amount of DNA released before and after inhibitor treatment (Fig. S1A).

The involvement of PAD4 in our model was suggested by chloroamidine treatment^18^, which inhibited 54% and 61% of NET release by *Leishmania*- and PMA-activated neutrophils, respectively (Fig. 1B). Regardless the donor, chloroamidine inhibited NET release induced by *Leishmania* in all experiments (Fig. S1B).

The involvement of PAD4 and elastase in the netosis triggered by *Leishmania* was further suggested by fluorescence microscopy. Neutrophils were unable of releasing NETs when pretreated with chloroamidine and elastase inhibitor as observed by the lack of NET-DNA staining in the presence of these inhibitors (Fig. S2).

Myeloperoxidase inhibition did not affect NET formation by *Leishmania*-activated neutrophils (Fig. 1C). However, myeloperoxidase inhibitor^19^ (300 nM) reduced 36% of NET release by PMA-activated neutrophils (Fig. 1C). This result is consistent with previous reports showing that myeloperoxidase is involved in NET formation induced by PMA^11^,^12^. Of note, chloroamidine, elastase and myeloperoxidase inhibitors and diphenyleneiodonium (DPI) were not toxic to neutrophils (Table S1).

### *Leishmania* promastigotes promote redox imbalance in neutrophils

**Figure 2:**
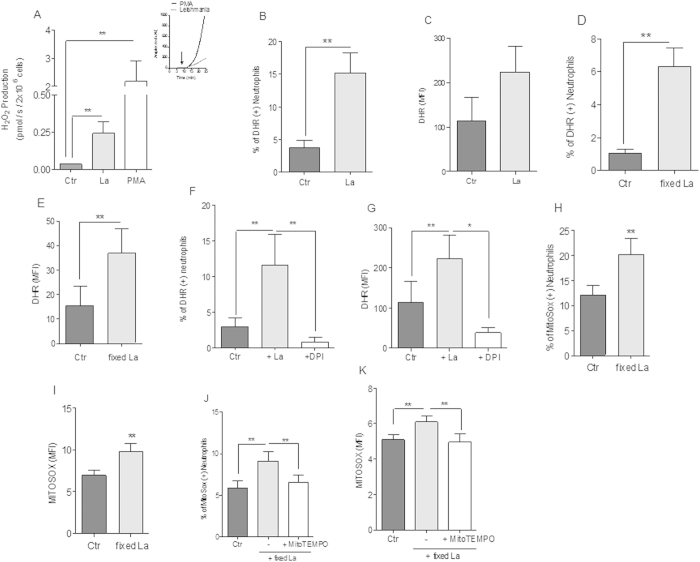
*Leishmania amazonensis* (La) promastigotes activate ROS production in human neutrophils. (**A**) H_2_O_2_ production was measured with Amplex Red (5 μM) after neutrophils (NØ; 2 × 10^6^) were stimulated with PMA (100 nM) or fixed promastigotes (1 NØ: 5 La ratio), and the fluorescence recorded over 25 min of incubation, as shown on inset. Data shown as mean ± SEM of 9 independent experiments were expressed as the amount of H_2_O_2_ produced in pmol/s/2 × 10^6^ neutrophils and as Amplex Red arbitrary units (AU, Inset). (**B–G**) Neutrophils (10^6^) were incubated with DPI (32 μM) for 30 min, and then stimulated with live (1NØ: 0.1La ratio) or fixed promastigotes (1NØ: 1 La ratio), incubated with DHR 123 (1.2 μM) for 20 min and analyzed by flow cytometry. Data are expressed as the percentage of DHR positive cells (**B,D,F**), and as the mean fluorescence intensity (MFI) (**C,E,G**) are shown as mean ± SEM of 23 (**B,C**), 4 (**D,E**) and 6 (**F,G**) different donors. Neutrophils (10^6^) were incubated with MitoSox Red probe (2.5 μM) for 30 min at 35 °C, 5% CO_2_, cells were washed and stimulated with fixed promastigotes (1NØ: 1 La ratio, **H,I**). Neutrophils (10^6^) were also treated with MitoSox Red (2.5 μM) and MitoTEMPO (100 μM, (**J,K**)). In both cases cells were then incubated for 30 min at 35 °C, 5% CO_2_ and analyzed by flow cytometer. Data are expressed as the mean ± SEM fluorescence intensity (MFI) of 20 (**H**) and 12 (**J**) donors. Data are expressed as the mean ± SEM percentage of MitoSox positive cells and (**I,K**) are shown as mean ± SEM of 11 donors. *p < 0.0001 and **p < 0.05.

The exposure of neutrophils to hydrogen peroxide (H_2_O_2_) induces H3 histone deimination mediated by PAD4, as previously described^20^. Since our results suggested the implication of histone deimination on classical netosis induced by *Leishmania* (Fig. 1B), we next investigated whether promastigotes would affect neutrophil redox metabolism. Thus, we followed the fluorescence increments of the redox-sensitive probe Amplex red coupled to horseradish peroxidase, which is specific for H_2_O_2_ quantification^21^. Our results show that, upon *Leishmania* or PMA-induced activation, gradually increasing levels of H_2_O_2_ were detectable within minutes after neutrophil challenge (Fig. 2A, *inset*). Indeed, in the presence of *Leishmania*, neutrophils significantly increased H_2_O_2_ production (Fig. 2A), as well as intracellular ROS levels (Fig. 2B–G). The observed redox imbalance occurs independently of parasite viability, as increased frequency of positive cells stained by dihydrorhodamine (DHR), and its fluorescence intensity, were observed in neutrophils stimulated by both viable (Fig. 2B,C) and paraformaldehyde-fixed *Leishmania* (Fig. 2D–G). Interestingly, DPI a flavoenzyme inhibitor^22^ impaired the boost in ROS formation induced by *Leishmania*, as evidenced by the low frequency of DHR positive cells and the fluorescence intensities as well (Fig. 2F,G). This suggests that *Leishmania* induced ROS production could occur through a NADPH oxidase-dependent mechanism, similarly to previous evidenced for PMA-induced neutrophil activation^9^.

Since *Leishmania* promastigotes were able to induce ROS production in neutrophils, we then investigated the potential contribution of mitochondria in this process. Mitochondrial ROS production was quantified by measuring the fluorescence signal of MitoSOX Red, a permeable dye that selectively targets mitochondria in live cells^23^. In order to determine only the neutrophil mitochondrial ROS production, the experiments were done with paraformaldehyde-fixed promastigotes. Our results show that *Leishmania* interaction with neutrophils induced a significantly higher frequency (Fig. 2H) and fluorescence intensity of positive MitoSOX Red stained neutrophils (Fig. 2I) when compared to control cells (20.2 *vs.* 12.1% positive MitoSOX Red stained cells, Fig. 2H; 9.8 *vs.* 6.9 AU, Fig. 2I). In order to confirm that promastigotes were stimulating mitochondrial ROS production, neutrophils were pretreated with MitoTEMPO, a mitochondrial-targeting antioxidant^23^. Pre-incubation of neutrophils with 100 μM MitoTEMPO before *Leishmania* interaction abrogated the increase in MitoSOX Red frequency of positive cells (Fig. 2J) and fluorescence intensity (Fig. 2K) induced by promastigotes (9.1 *vs*. 6.6% positive MitoSOX Red stained cells, Fig. 2J; 6.1*vs* 4.9 AU, Fig. 2K). MitoTEMPO was not toxic or induced apoptosis to neutrophils in the conditions used in the assays (Fig. S3). These data clearly demonstrate that *Leishmania* interaction with neutrophils induced mitochondrial ROS production.

### Role of reactive oxygen species, Nitric oxide synthase, Mitochondrial ROS and xanthine oxidase on *Leishmania*-induced classical netosis

**Figure 3:**
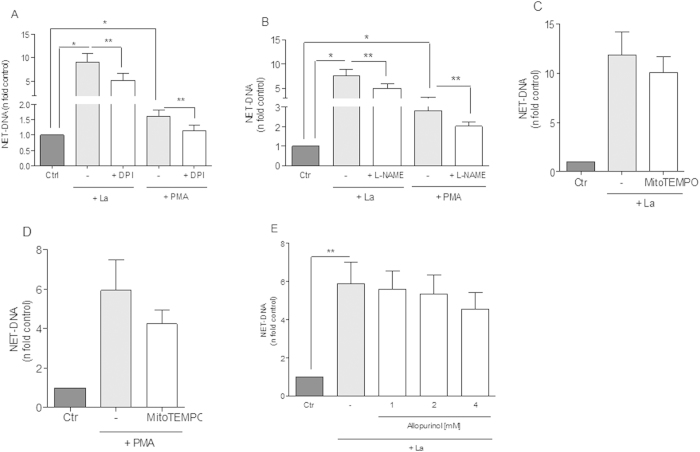
ROS and RNS role in NET release induced by *Leishmania amazonensis* (La). Neutrophils (NØ; 2 × 10^6^) were incubated with (**A**) DPI (32 μM), (**B**) L-NAME (1 mM), (**C,D**) MitoTempo (100 μM) and (**E**) Allopurinol (1, 2 and 4 mM) for 30 min and then stimulated or not with promastigotes (1 NØ: 0.1La ratio) or PMA (100 nM) for 1 h, followed by DNA quantification using PicoGreen assay kit in culture supernatants. Data were normalized according spontaneous release of NET-DNA representing the mean ± SEM from 22 (**A**), 12 (**B**), 7 (**C**), 11 (**D**), 10 (**E**) and 11 (**F**) donors. *p < 0.0001 and **p < 0.05.

Following, we investigated the mechanisms responsible for ROS production that contribute to *Leishmania*-induced NET formation. Initially we tested the capacity of the classical NET inhibitor DPI^2^, to inhibit netosis induced by *Leishmania* promastigotes. In our experimental conditions, DPI significantly inhibited 43% of netosis induced by *Leishmania*. In concurrent assays using PMA as inducer, netosis inhibition was also seen after neutrophils treatment with DPI (Fig. 3A). NET-released after parasite stimulus varied among donors, but DPI significantly decreased netosis induced by *Leishmania* as observed by reduced NET-DNA concentration released (Fig. S1D) and by fluorescence microscopy (Fig. S2). Likewise, pretreatment of neutrophils with apocynin, which inhibits NADPH oxidase^24^, significantly decreased NET release induced by *Leishmania* (Fig. S1E). Altogether, our results suggest that *Leishmania* induced netosis requires, at least partially, ROS derived from a functional NADPH oxidase complex.

Neutrophils produce and release nitric oxide (NO) spontaneously or following activation and both isoforms of NO synthase (NOS), inducible and constitutive, have been purified from human neutrophils^25^,^26^. To assess NO participation on *Leishmania* induced netosis we treated neutrophils with L-NAME, a classical NOS inhibitor^27^. Our results showed that L-NAME significantly inhibited 33.5% of NET-release stimulated by *Leishmania* and 27% by PMA (Fig. 3B).

Although mitochondria seems to contribute little to bulk oxygen consumption in neutrophils^28^,^29^,^30^, interference on electron transport system (ETS) at complexes I^31^ and III^32^ cause significant increase on ROS generation in these cells. Since *Leishmania* promastigotes were able to induce mitochondrial ROS production in neutrophils (Fig. 2), we investigated the potential contribution of this source for netosis. Scavenging mitochondrial ROS caused no apparent effect on both *Leishmania* (Fig. 3C) and PMA-induced netosis (Fig. 3D). These data demonstrate that despite *Leishmania* induced neutrophil mitochondrial ROS production, parasite-induced netosis occurs independently of this process. No toxicity was observed for neutrophils nor for parasites by MitoTempo at the concentrations used (Table S1, Fig. S3).

Next, we tested if xanthine oxidase could modulate netosis pretreating neutrophils with its inhibitor, allopurinol^33^. Our results demonstrated that allopurinol did not inhibit NET release induced by parasites (Fig. 3E). Interestingly, inhibition of xanthine oxidase (Fig. 3E) or myeloperoxidase (Fig. 1C) caused no effect on *Leishmania*-induced netosis, indicating the involvement of selective redox-dependent mechanisms in this process. Despite this general trend, inhibition of myeloperoxidase decreased *Leishmania*-induced NET release in some donors (Fig. S1C). Noteworthy, NET release promoted by PMA was significantly reduced (34%) by myeloperoxidase inhibitor, in agreement with previous evidence in the literature^9^,^12^.

### *Leishmania* promastigotes activate an early/rapid netosis mechanism

**Figure 4:**
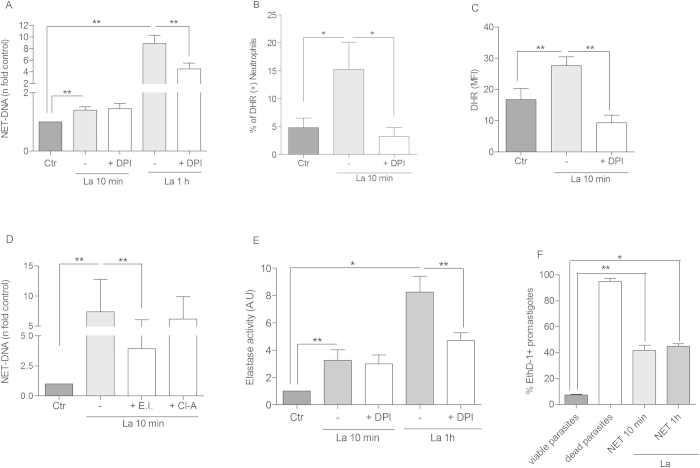
Rapid/Early netosis stimulated by *Leishmania amazonensis* (La) promastigotes occurs in a ROS-independent manner. (**A**) Neutrophils (2 × 10^6^) were incubated with DPI (32 μM) for 20 min and then, stimulated or not with promastigotes (1 NØ: 0.1 La ratio) for 10 min and 1 h. Following stimulation, DNA quantification of samples was performed using PicoGreen. (**B,C**) Neutrophils (10^6^) treated with DPI (32 μM) for 20 min, were stimulated with promastigotes (1 NØ: 0.1La ratio) for 10 min, incubated with DHR 123 (1.2 μM) and immediately analyzed by flow cytometry. (**D**) Neutrophils (NØ; 2 × 10^6^) were incubated with elastase inhibitor (Ei, 10 μM) and chloroamidine (Cl-A, 12 μM) for 20 min and then, stimulated with promastigotes (1NØ: 0.1 La ratio) for 10 min. DNA quantification was done as above. (**E**) Supernatants obtained from 10 min early/rapid or 1 h classical NET induction protocol were incubated with the elastase substrate, N-methoxysuccinyl-Ala-Ala-Pro-Val-7-amido-4-methylcoumarin (0.25 mM) for 30 min at 37 °C, 5% CO_2_ and the reaction product analyzed at 360/455 nm. (**F**) *Leishmania* killing after parasite treatment with NET-rich supernatants. Promastigotes (1 × 10^6^) were incubated or not during 2 h with NET-rich supernatants obtained after 10 min or 1 h of stimuli. Then, parasites were stained with ethidium homodimer-1 (EthD-1) for 30 min and analyzed by flow cytometry. The positive control (dead parasites) was done with promastigotes treated with 0.1% Triton X-100, followed 3 cycles of freeze and thaw. Results are shown as the mean ± SEM of 3 independent experiments. *p < 0.0001 and **p < 0.05. (**A,D**) Data were normalized regarding spontaneous release of DNA representing the mean ± SEM from 7 (**A**) and 9 (D) different donors. (**B,C**) Data expressed as the percentage of DHR positive cells (**B**), and as the mean fluorescence intensity (MFI) (**C**) are shown as mean ± SEM of 7 different donors. (**E**) Data are expressed as the mean ± SEM of elastase activity in arbitrary units (AU) from 5 different donors. *p < 0.02 and **p < 0.05.

It has been shown that *Staphylococcus aureus* induces an early/rapid NET release, through a ROS independent pathway^5^. Thus, we sought to verify whether *Leishmania* would induce a similar mechanism. Indeed, promastigotes induced NET release after 10 min incubation with neutrophils, and this early/rapid induction was not affected by DPI, which contrasts with the inhibitory role of this compound on the classical netosis testing the same donors (Fig. 4A). Additionally, these results were confirmed by immunofluorescence staining of NETs released at 10 min, which were not inhibited by DPI (Fig. S4). Interestingly, *Leishmania* induced ROS production detected by the frequency of DHR positive cells and by its fluorescence intensity at this early time point was significantly decreased by DPI treatment (Fig. 4B,C). The percentage of cells undergoing early NET release was 29% and increased to 41% after 1 h post stimuli (Fig. S5). Additionally, Amplex red poorly stained 10 min NETs, but strongly labeled 1 h NETs (Fig. S6), showing a higher oxygen peroxide content after 1 h of NET induction by *Leishmania*. This early/rapid NET release mechanism was inhibited by the elastase inhibitor but, differently from the classical netosis, was unaffected by chloroamidine (Fig. 4D). NETs formed at 10 min and 1 h were equally stained for histone H3 citrullination, but the addition of chloroamidine lowered the histone citrullination labeling only after 1 h of NET induction (Fig. S7). Because the chloroamidine inhibition also reduced NET formation only after 1 h induction (Fig. 1B), we could suggest that the contribution of PAD4 activity to NET release is relevant only during classical netosis. Furthermore, we found that the elastase activity detected in the early/rapid mechanism, it is not inhibited by DPI (Fig. 4E), in contrast with the decreased enzyme activity promoted by DPI in the classical netosis (Fig. 4E).

We further evaluated the promastigote viability after parasite treatment with NET-rich supernatants obtained by early/rapid or classical netosis, and found 42% and 45% reduction in the *Leishmania* survival, respectively, indicating that NETs released by the early/rapid mechanism possess the same leishmanicidal efficiency as NETs released by the classical mechanism (Fig. 4F).

## Discussion

We described here that *L. amazonensis* promastigotes induce neutrophil redox imbalance and NET release, involving two kinetically and mechanistically distinct processes: an early redox-independent, occurring only ten minutes after parasite contact, followed by a later redox-dependent process, reliant on ROS/RNS generated by NADPH oxidase complex and NO synthase activities. Also, netosis induced by these parasites requires the involvement of active neutrophil elastase and PAD4, but not MPO. Together, our data implicates the dynamic control of neutrophil redox homeostasis as an important mechanism of *Leishmania*-induced netosis.

One of the hallmarks of netosis is chromatin decondensation, which depends on both elastase and PAD4 activities and to an unknown mechanism mediated by MPO^11^,^12^,^13^,^14^,^15^. The role of elastase was evidenced by a decrease in netosis after inhibition of its enzymatic activity^11^. Moreover, elastase knockout mice were unable to release NETs after a *Klebsiella pneumoniae* infection^11^. We demonstrate that *Leishmania*-induced netosis is dependent on elastase activity, since its inhibition significantly affected NET release. Similarly, evidence from the literature have pointed out that PMA-stimulated netosis in neutrophils was also repressed by elastase inhibition^11^.Interestingly, the inhibition of NADPH oxidase activity by DPI produced kinetically distinct results both on elastase activity and on NET release, which may be explained by the different mechanisms that contribute to redox homeostasis at early (antioxidant levels) and later (NADPH oxidase + NO synthase) stages of interaction.

Histone citrullination by PAD4 is a fundamental step for chromatin decondensation and NET extrusion^13^,^14^,^15^. In this sense, we demonstrated that PAD4 inhibition reduced NET release by *Leishmania*-stimulated neutrophils, implicating this enzyme in parasite-induced classical netosis. Similar, PMA-stimulated netosis was also reduced by PAD4 inhibition. However, it remains elusive whether there is a direct relationship between protein citrullination, PAD4 activity and redox metabolism. Evidence from the literature demonstrates a boost in arginine conversion to citrulline when bone marrow cells were incubated in the presence of leukocyte conditioned medium and a superoxide radical generation source^34^. Interestingly, citrulline production in this system seems to be arginine deiminase and PAD4-independent, as their activities were detected in the assays conditions.

It has been shown that MPO synergize the effect of elastase on chromatin decondensation during netosis^11^. Neutrophils from MPO deficient donors were unable to release NETs when stimulated by PMA or *Candida albicans*^12^. However, the role of MPO on netosis seems to depend on the nature of the stimulus, since MPO-deficient neutrophils release NETs when stimulated with *P. aeruginosa, S. aureus* or *E. coli*, but fail to respond to PMA^35^. Likewise, inhibition of MPO activity in normal neutrophils had no effect on NET induction by *P. aeruginosa*, but decreased PMA-triggered netosis^35^. Interestingly, species-specificities also exist in terms of NET formation since inhibition of mice neutrophil MPO prevented *Pseudomonas*-induced netosis^36^, which is in contrast to netosis induced by the same pathogen in human neutrophils^37^. We demonstrated that *Leishmania*-induced netosis is not dependent on MPO activity, a feature that contrasts with netosis promoted by PMA and *Candida albicans*^12^, but not by other pathogens^35^. Interestingly, NO-mediated netosis is somehow dependent of active MPO, as pharmacological inhibition of this enzyme abrogates NET release^38^.This seems not to be the case in our experimental setting, since *Leishmania*-induced netosis is partially dependent on active NO synthase, but independent of MPO.

To address the role of ROS on *Leishmania*-induced netosis we investigated different sources of these species during neutrophil-promastigote interaction. Our results point out that NOS is involved in *Leishmania*-induced NET release, since L-NAME significantly decreased netosis by the parasite and by PMA as well. Indeed, inducible NO synthase activity is frequently detected at inflammation and infection sites and has already been demonstrated in cutaneous leishmaniasis lesion^39^,^40^,^41^,^42^. It has been reported that NO induces NET release from human neutrophils, which may also synergize with other stimuli present in the infection site, increasing netosis^38^, and NETs were evidenced in human cutaneous leishmaniasis biopsies^3^,^43^.

It is worth noting that, whatever the exact mechanism, *Leishmania* and PMA-induced classical netosis, several features were shared, as both are dependent on elastase, PAD4, NO synthase and NADPH oxidase activities, and independent of mitochondrial-derived ROS production. In this regard, despite neutrophils mitochondria^44^ contributes little to cellular oxygen consumption^29^, inhibition of electron transport system at complexes I^31^ and III^32^ significantly increased ROS generation, indicating the potential of this organelle to modulate cellular redox homeostasis. Remarkably, although *Leishmania* interaction increased mitochondrial ROS production in human neutrophils, scavenging this ROS source had no effect on NET-release. In line with our observations, previous evidence demonstrated that interference of mitochondrial ROS generation, by the use of protonophores, caused no effect on PMA-induced netosis^9^. Evidences presented here indicate that netosis induced by *Leishmania* require core specific neutrophil processes, regardless the stimuli, which do not involve mitochondrial-derived ROS.

The potential contribution of xanthine oxidase on *Leishmania*-induced netosis as another source of cellular ROS was investigated. By using the classical xanthine oxidase inhibitor allopurinol, we observed that it caused no effect on NET production stimulated by the parasite.

Considering that the contribution of NADPH oxidase-derived ROS to the classical netosis has been largely demonstrated in the literature^4^,^45^, we tested this source of ROS, initially using pharmacological inhibitors. In our model, the association between *Leishmania*-induced classical netosis and ROS production by NADPH oxidase was suggested, since pretreatment of neutrophils with apocynin and DPI, inhibited NET extrusion induced by the promastigotes. Contrarily, it has been reported that *L. donovani,* a species that cause visceral leishmaniasis, induces netosis in a ROS-independent way^46^. This discrepancy with our results could be due to the different species of the parasite assayed or, most likely, because of the higher parasite-to-neutrophil ratio used in the *L. donovani* study. In our model, netosis inhibition by DPI was not observed when a one parasite per neutrophil ratio was assayed (data not shown).

Although the majority of NET inducers require ROS generation by NADPH oxidase, there are reports of ROS-independent netosis induced by *Staphylococcus aureus, Candida albicans,* uric acid, MIP-2 and ionomycin^5^,^35^,^47^,^48^,^49^. Moreover, netosis induction on carp granulocytes rely on ROS production when stimulated by PMA and polyinosinic:polycytidylic acid, but is ROS-independent when lipopolysaccharide was the stimulus^50^. It has also been described that *S. aureus* induces NET release by an alternative mechanism, which is ROS independent and occurs in a rapid/early period of time^5^,^6^. Interestingly, we observed that only 10 minutes after *Leishmania* interaction, neutrophils promote ROS generation by NADPH oxidase activity and NET release. However, early *Leishmania*-induced NET release is independent of ROS generation since treatment with DPI caused no effect on early NETs formation, contrasting to classical netosis.

It has been already demonstrated that NETs are toxic to several microorganisms, such bacteria and fungi^1^,^4^,^51^, and we also reported that *L.amazonensis* promastigotes induce and are killed by NETs released by the presently designated classical mechanism^6^. In this work we reveal that NETs released by early/rapid mechanism present the same leishmanicidal efficiency as those extruded by the classical mechanism, indicating that NETs are able to reduce parasite survival regardless their induction mechanism.

Similarly to NET release stimulated by *S. aureus,* our present results demonstrated that *Leishmania* could trigger the two types of netosis described: (*i*) an early/rapid ROS-independent and (*ii*) a late ROS-dependent mechanism^6^. Thus, *Leishmania* triggers netosis by a ROS-dependent pathway after about 1 hour, as well as by a ROS-independent mechanism occurring in 10 minutes after initial contact. Conceivably, redox imbalance promoted by *Leishmania*-neutrophil interaction would contribute to improve parasite trapping by NET-released DNA and their subsequent killing by neutrophil histones^3^.

Based on the data presented here, it is conceivable that promotion of neutrophil ROS formation, through the activation of pro-oxidant mechanisms, may improve parasite killing *in vivo*. Further studies are required to better understand how neutrophil redox mechanisms triggered by *Leishmania* induced NET formation, the dynamics of this process and their consequences for the host immune system and potential as therapeutic target.

## Methods

### Neutrophil purification

after informed consent from donors, blood was obtained and neutrophils were isolated by density gradient centrifugation (Histopaque; Sigma) followed by hypotonic lysis of erythrocytes. Purified neutrophils (≥95% of the cells) were re-suspended in RPMI 1640 medium (Sigma) and kept on ice until use. Experiments with human cells were performed in full compliance with the guidelines of the Research Ethics Committee of the Hospital Universitário Clementino Fraga Filho (Comite de Ética em Pesquisa (CEP), Universidade Federal do Rio de Janeiro, Brazil), and approved under the number 055-15.

### Parasites

*Leishmania amazonensis* (MHOM/BR/75/Josefa) was maintained in Schneider Insect’s medium (Sigma) supplemented with 10% heat inactivated fetal calf serum and 40 μg/ml of gentamicin at 26 °C. Stationary phase promastigotes of 5–6 day cultures were collected, washed three times in PBS and resuspended in RPMI for further use. For some experiments promastigotes were fixed in 4% paraformaldehyde for 1 h, room temperature, extensively washed with PBS (20 ml, 4 times, 2760 g) and resuspended in RPMI.

### NETs Inhibition Assays

Neutrophils (2 × 10^6^, 200 μl) were incubated with the following inhibitors for 30 min at 35 °C, 5% CO_2_: diphenyleneiodonium (DPI; 32 μM; Sigma), Apocynin (APO; 1 μM; Sigma), MitoTEMPO (100 μM; Santa Cruz Biotech), N (G)-nitro-L- arginine methyl ester (L-NAME; 1 mM, Sigma), chloroamidine (Cl-A; 12 μM; Cayman Chemical), elastase inhibitor III (E.i; 5 or 10 μM, MeOSuc-Ala-Ala-Pro-Val-CMK; Calbiochem) or myeloperoxidase inhibitor-1 (MPOi; 300 nM; Calbiochem). Next, stimuli were added (promastigotes at 0.1 parasite: 1 neutrophil ratio or PMA (100 nM; Merck), and the culture incubated at 35 °C, 5% CO_2_ for 1.5 h. Supernatants were collected for DNA quantification using dsDNA Picogreen kit (Invitrogen), as described^3^. Early NET induction was performed as above except that neutrophils were incubated with the parasites for only 10 min. ROS-dependency of this early mechanism was carried out by pre-treating neutrophils with DPI (32 μM) for 20 min at 35 °C, 5% CO_2,_ before adding the promastigotes. The role of elastase and PAD4 in the early mechanism was also evaluated treating neutrophils as above with chloroamidine and elastase inhibitor.

### Recovery of NET-rich supernatants

Neutrophils (2 × 10^6^, 200 μL) were incubated with *Leishmania* at 0.1 promastigotes: 1 neutrophil ratio during 10 min or 1 h at 35 °C, 5% CO_2_. Then, NET-rich supernatants were collected after cell culture centrifugation at 400 g for 10 min, followed by a second supernatant centrifugation at 2760 g for 20 min, to remove parasites. NET-rich supernatants were used immediately after production.

### Parasite survival assay

Promastigotes (10^6^, 200 μL) were incubated with NET-rich supernatants during 2 h at 35 °C, 5% CO_2_, and cell viability was assessed by treating parasites with 4 μM ethidium homodimer-1 (EthD-1) staining solution for 30 min, according to the manufacturer’s instructions (Molecular Probes). Promastigotes killed by 0.1% Triton X-100 (Sigma) treatment, followed by 3 cycles of freeze and thaw, served as positive control. Data were collected in a FACSCalibur flow cytometer, and analyzed with Summit v4.3 software.

### ROS Production

Neutrophils (10^6^, 200 μL) were pre-incubated or not with DPI (32 μM) for 30 min at 35 °C, 5% CO_2_. Afterwards, dihydrorhodamine123 (DHR, 1.2 μM, Sigma) was added, and neutrophils were stimulated with promastigotes (10^5^) or with PMA (100 nM), for 15 min at 35 °C, 5% CO_2_. The fluorescence intensity of individual cells was analyzed with a FACSCalibur flow cytometer. Data analyses were performed with Summit v4.3 software.

### Quantification of hydrogen peroxide production rate

H_2_O_2_ production by neutrophils (2 × 10^6^, 200 μL), was measured with Amplex Red (Invitrogen) after stimulation with PMA (100 nM) or fixed promastigotes (10^7^). H_2_O_2_ production was assessed by monitoring resorufin fluorescence due to the oxidation of 5 μM Amplex Red in the presence of 20 mg/mL horseradish peroxidase (Sigma), in the buffer: 125 mM NaCl, 3 mM KCl, 2 mM CaCl_2_, 1.25 mM NaH_2_PO_4_, 2 mM MgCl_2,_ 20 mM HEPES. The rate of Amplex Red oxidation was recorded at room temperature using a Cary Eclipse spectrofluorimeter (Varian) adapted with a continuous stirring device, operating at excitation and emission wavelengths of 530 nm and 590 nm, respectively. After each measurement, a standard curve of H_2_O_2_ reagent grade (Sigma) was performed.

### Mitochondrial ROS production

Neutrophils (10^6^, 200 μL) were pre-incubated with MitoSox-Red (2.5 μM, Invitrogen) for 30 min at 35 °C, 5% CO_2_. Next, cells were washed and stimulated with fixed promastigotes (10^6^) for 30 min at 35 °C, 5% CO_2_. Control experiments were carried out with neutrophils pre-incubated with MitoTEMPO (100 μM) and further challenged with promastigotes in the same conditions as described above. The optimum concentration of modulators of cellular ROS production and mitochondrial function were determined by titration of the individual compounds against human neutrophils in separate experiments. The fluorescence intensity of individual cells was analyzed by flow cytometry in a FACSCalibur flow cytometer. Data analyses were performed on Summit v4.3 software.

### Immunofluorescence

Neutrophils (10^5^, 300 μL) adhered to 0.001% poly-L-lysine-coated slide were incubated with or without inhibitors, cultured with promastigotes (10^5^) for 10 min or 1 h as described above, and fixed in 4% paraformaldehyde. NETs were visualized in a Zeiss Axioplan microscope after staining with propidium iodide (PI, 400 nM, Invitrogen). Labeling of citrullinated H3 histone was done with anti-Histone H3 (1:800; citrulline R2 + R8 + R17; AB5103, Abcam), for 1 h, followed by secondary antibody goat-anti-rabbit conjugated to Alexa 488 (1:800, Molecular Probes) together with DAPI (10 μg/mL, Sigma) for 30 min. To visualize netosis and peroxide formation, neutrophils (10^5^) and promastigotes (10^4^) were prepared alive in the presence of Amplex Red (5 μM, Molecular Probes) and Sytox green (0.5 μM, Invitrogen) and photographed after 10, 40 or 60 min of incubation. Images were taken using Leica DMI 6000 microscope.

### Elastase activity assay

Supernatants (25 μL) obtained from early NET induction protocol were incubated with the elastase substrate, N-methoxysuccinyl-Ala-Ala-Pro-Val-7-amido-4-methylcoumarin (0.25 mM, Sigma) in buffer (50 mM HEPES, 100 mM NaCl and 0.01% Triton X-100) for 30 min at 37 °C, 5% CO_2_ protected from light. The reaction product was analyzed at 360/455 nm.

### Apoptosis assessment

Neutrophils (10^6^, 200 μL) were suspended in Annexin V (AnV) binding buffer (10 mM HEPES, 150 mM NaCl, 2.5 mM CaCl_2_) at pH 7.2, treated with MitoTEMPO (100 μM) for 30 min at 35 °C, 5% CO_2_ and then incubated at room temperature for 15 min with AnV-FITC (Molecular Probes) as indicated by the manufacturer. Apoptotic neutrophils were obtained by exposing cells to UV light for 2 h. Cells were analyzed by flow cytometry using a FACSCalibur flow cytometer.

### Cytotoxicity of inhibitors to neutrophils and parasites

Neutrophils (2 × 10^6^, 200 μL) were incubated with the different inhibitors for 2 h in the same conditions used for the assays, and supernatants were collected for cell viability evaluation using the CytoTox® kit (Promega). Tween lysed neutrophils and purified lactate dehydrogenase were used as positive controls. Promastigotes (10^6^) were incubated for 1 h with supernatants collected from neutrophils treated with the different inhibitors in the same conditions used for the assays. Then, PI (100 μg/ml; Sigma) was added immediately before reading on a FACSCalibur flow cytometer.

### Quantification of neutrophils nuclei morphology

Neutrophils (10^5^, 300 μL RPMI) were seeded on 0.001% poly-L-lysine-coated glass coverslip inside a 24 well plate and incubated with *L. amazonensis* promastigotes (10^4^) for 10 and 60 min. Cultures were fixed with 4% paraformaldehyde and stained with propidium iodide (PI, 400 nM, Invitrogen) for 10 min and gently washed twice with PBS. Images were captured using a Zeiss Axioplan-2 microscope (Oberkochen, Germany) equipped with a Color View XS digital video camera. The nuclear shape (lobulated or decondensed) of 150–400 cells from three different donors was counted using Image J 1.46r software (National Institutes of Health), and the results shown as percentage of condensed or decondensed (netosis) neutrophils.

### Statistical Analyses

Data were presented as mean ± SEM values for at least 4 different replicates of independent experiments. D´Agostino and Pearson normality tests were done for all values to assess their Gaussian distribution. Comparisons between groups were done by one-way ANOVA and a posteriori Tukey’s test for pair-wise comparisons. When appropriate, unpaired Student’s t-tests or Mann-Whitney´s test were employed. Differences of p < 0.05 were considered to be significant. When Gaussian distribution was achieved, outlier values were excluded by performing the Grubbs’ test using the online tool available at http://graphpad.com/quickcalcs/Grubbs1.cfm. All graphs and analyses were carried out by using the GraphPad Prism software version 5.00 for Windows (GraphPad Software, USA).

## Additional Information

**How to cite this article**: Rochael, N. C. *et al.* Classical ROS-dependent and early/rapid ROS-independent release of Neutrophil Extracellular Traps triggered by *Leishmania* parasites. *Sci. Rep.* **5**, 18302; doi: 10.1038/srep18302 (2015).

## Supplementary Material

Supplementary Information
