# Supplementary material for: Classical ROS-dependent and early/rapid ROS-independent release of Neutrophil Extracellular Traps triggered by Leishmania parasites
Source: Sci Rep. 2015 Dec 17;5:18302. doi: 10.1038/srep18302 (PMC4682131; doi:10.1038/srep18302)
Supplement: Supplementary Information [file srep18302-s1.pdf]

Classical ROS-dependent and early/rapid ROS-independent release of Neutrophil Extracellular Traps triggered by *Leishmania* parasites.

Natalia C. Rochael[1], Anderson B. Guimarães-Costa[1,4], Michelle T.C.Nascimento[1], Thiago S.DeSouza-Vieira[1], Matheus P.Oliveira[2,3], Luiz F. Garcia e Souza[2,3],Marcus F. Oliveira[2*], Elvira M. Saraiva[1*]

Table S1. Neutrophil viability measured by lactate dehydrogenase (LDH) assay*.

Treatment [Concentration]	% of Viable Neutrophils*
Elastase inhibitor [10 µM]	97.40 ± 0.99
Chloroamidine [12 µM]	92.99 ± 5.07
Allopurinol [4 mM]	99.99
DPI [32 µM]	97.36 ± 2.64
MitoTEMPO [100 µM]	99.97 ± 0.01
MPO inhibitor-1 [300 nM]	100
L-NAME [1 mM]	100

*Human neutrophils ($2x10^6$) were incubated with the different inhibitors for 2h in the conditions used for the assays, supernatants were collected and evaluated using the CytoTox® kit (Promega). Tween lysed neutrophils and purified lactate dehydrogenase were used as positive controls. Reaction reading was carried out at 490 nm. Data shown as % of viable neutrophils from 3 experiments.

Figure S1.

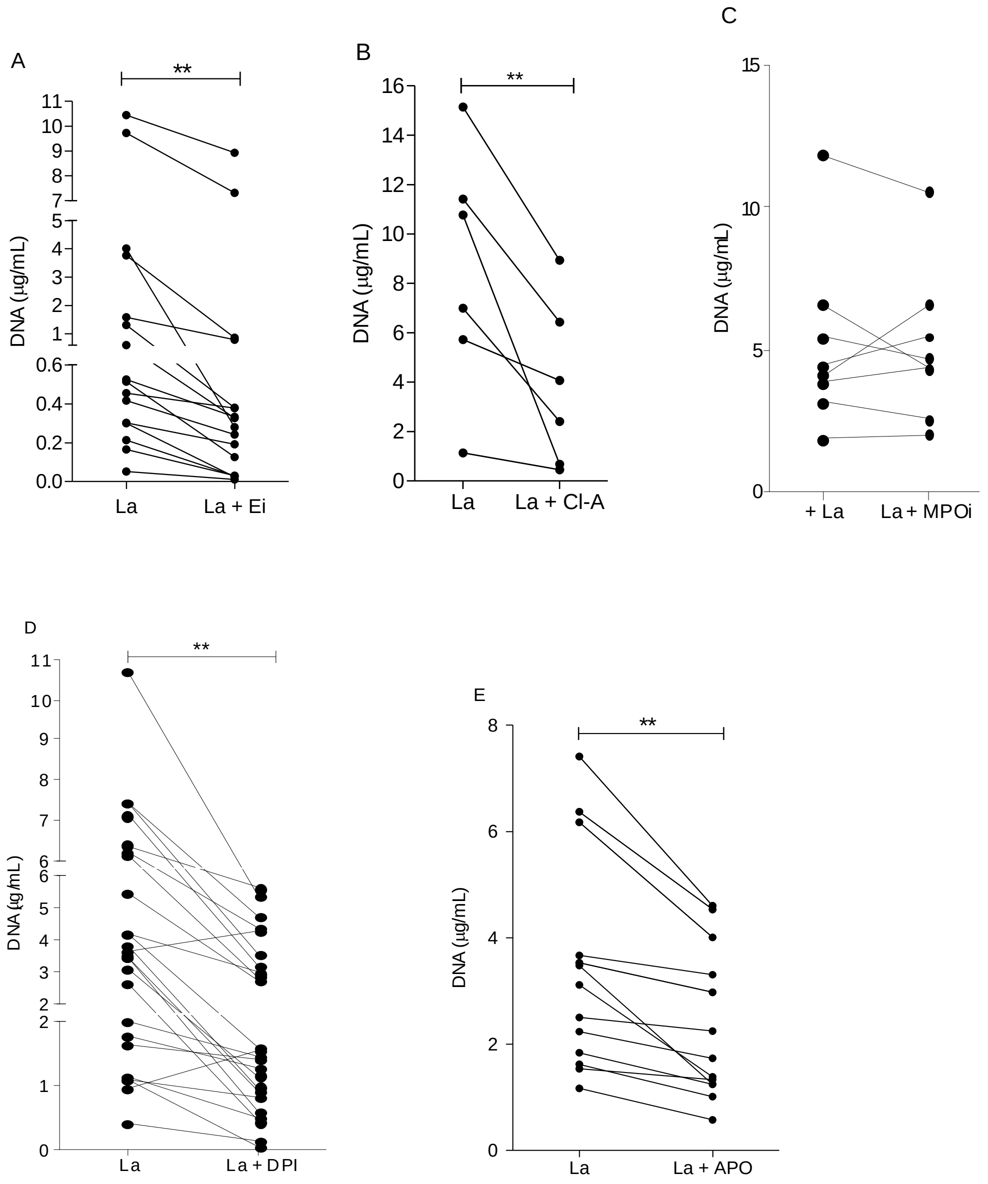

Figure S1. Donor-to-donor variation in the netosis induced by *Leishmania amazonensis* (La) promastigotes. Neutrophils (2 x 10^6) were incubated with (A) Elastase inhibitor (E.i, 10 µM), (B) Chloroamidine (Cl-A, 12 µM), (C) Myeloperoxidase inhibitor (MPOi, 300 nM), (D) Diphenyleneiodonium (DPI, 32 µM) and Apocynin (APO, 1µM) for 30 min and then, stimulated or not with promastigotes (1NØ: 0.1 La ratio) for 1h. Following stimulation, DNA quantification of samples was performed using PicoGreen assay kit in culture

supernatants. Data show the amounts of DNA (μg/ml) detected in the culture supernatants in La stimulated with or without the depicted inhibitors. **p<0.05.

Figure S2.

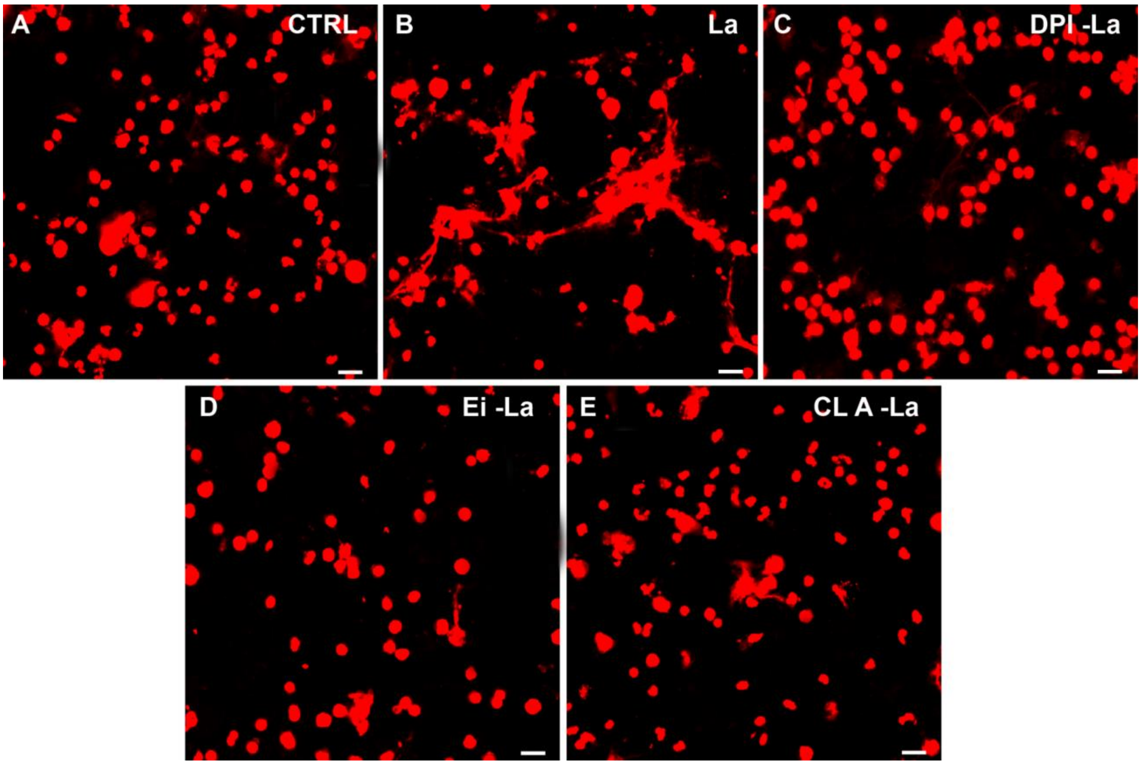

Figure S2. Immunostaining of classical NET release induced by *Leishmania amazonensis*. Neutrophils stimulated or not with *Leishmania* promastigotes (1:5 ratio) were treated or not with the indicated inhibitors and incubated for 1 h at 35°C. Cells were fixed and stained with PI. (A) Unstimulated control neutrophils; (B) neutrophils stimulated with promastigotes; (C) neutrophils treated with DPI (32 μM) and stimulated with promastigotes; (D) neutrophils treated with the elastase inhibitor (10 μM) and stimulated with promastigotes; (E) neutrophils treated with chloroamidine (Cl-A, 12 μM) and stimulated with promastigotes. Bars: 20 μm.

Figure S3.

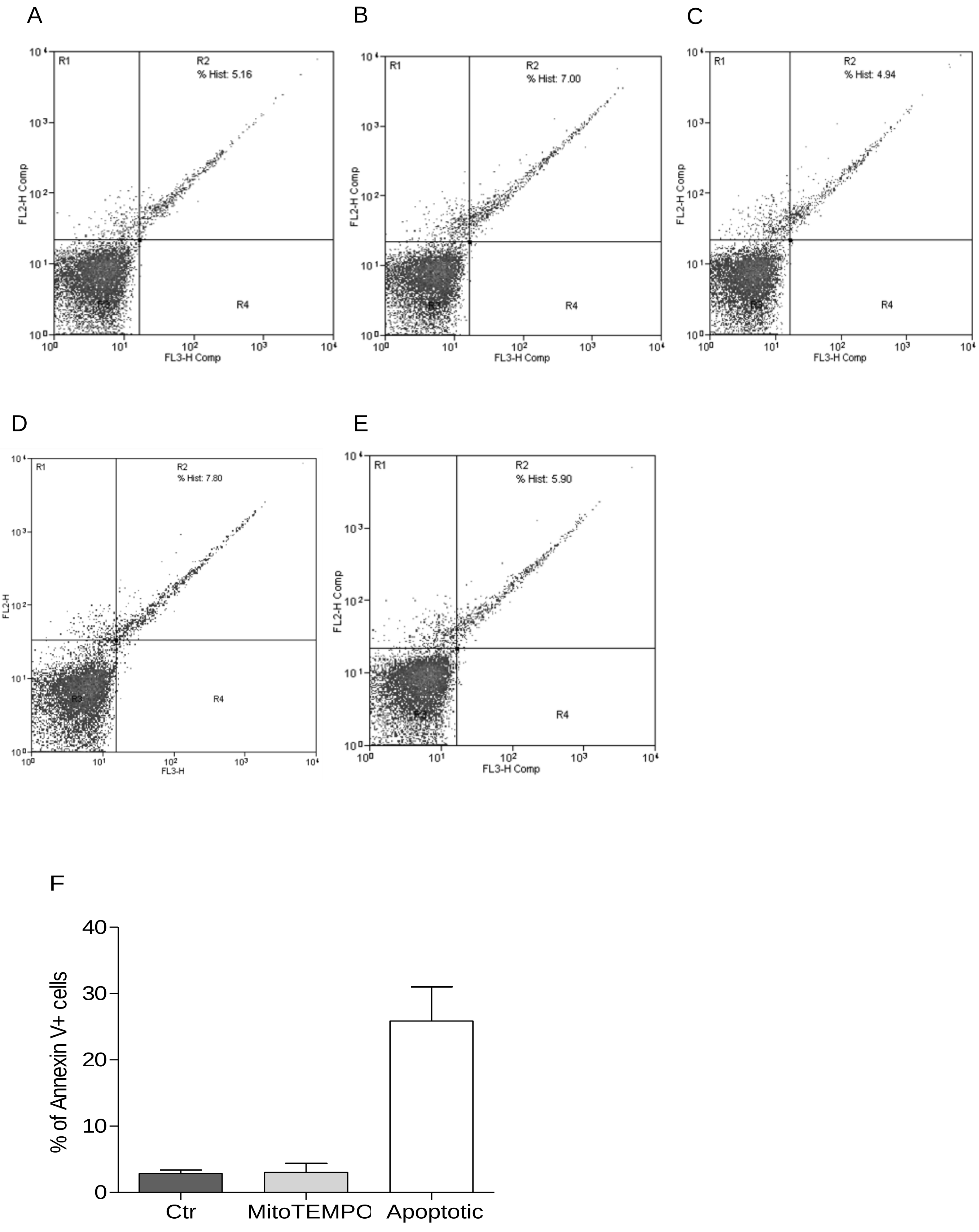
A
R1
R2
% Hist: 5.16
R4
FL2-H Comp
FL3-H Comp
B
R1
R2
% Hist: 7.00
R4
FL2-H Comp
FL3-H Comp
C
R1
R2
% Hist: 4.94
R4
FL2-H Comp
FL3-H Comp
D
R1
R2
% Hist: 7.80
R3
R4
FL2-H
FL3-H
E
R1
R2
% Hist: 5.90
R4
FL2-H Comp
FL3-H Comp
F
% of Annexin V+ cells
0
10
20
30
40
Ctr
MitoTEMPO
Apoptotic

Figure S3. Safety of inhibitors to the parasites. (A-E) Supernatants collected from neutrophils (10^6) stimulated with the different inhibitors for 30 min in the conditions used for the assays were collected and incubated with *Leishmania amazonensis* promastigotes (10^6) for 1h. One representative experiment out of two with similar results: Control (A), elastase inhibitor (B), Chloroamidine (C), DPI (D) and MitoTEMPO (E). Viability was determined by propidium iodide (PI, 100 µg/ml) labeling on a FACSCalibur flow cytometer. **(F) Mitochondria inhibitor did not induce apoptosis on human neutrophils**. Neutrophils treated with MitoTEMPO (100 µM) for 30 min at 35ºC, 5% CO_2 were then incubated at room temperature for 15 min with AnV-FITC (1:100) according to the manufacturer instructions. Neutrophils exposed to UV light for 2h were used as positive control. Cells were analyzed by flow cytometry.

Figure S4.

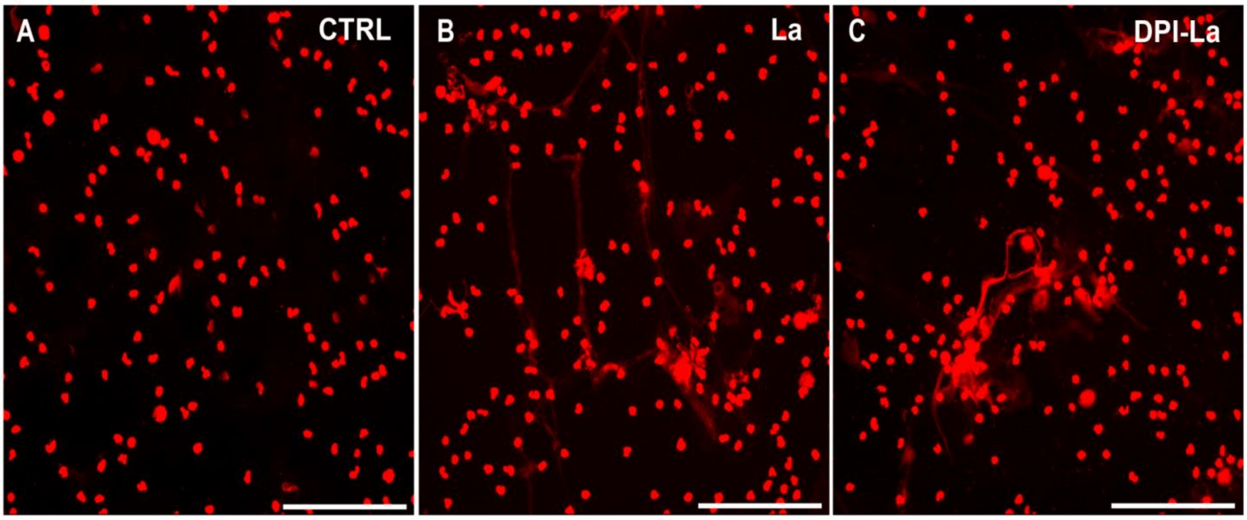

Figure S4. Immunostaining of the early/rapid NET release induced by *Leishmania amazonensis*. Neutrophils pretreated with DPI (32 µM) were incubated or not with promastigotes (1:5 ratio) for 10 minutes at 35°C. Cells were fixed with paraformaldehyde and stained with PI. (A) Unstimulated control neutrophils; (B) neutrophils stimulated with promastigotes; (C) neutrophils pretreated with DPI (32 µM) and stimulated with promastigotes. Bars: 50 µm.

Figure S5.

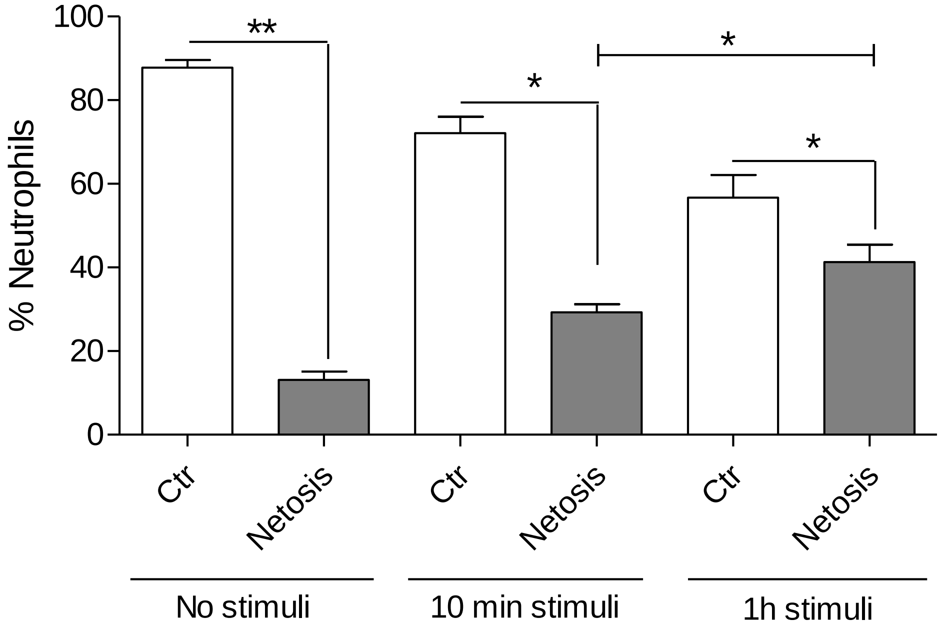

Figure S5. Quantification neutrophils' of nuclear morphology. Neutrophils (10^5) were stimulated with *Leishmania amazonensis* promastigotes (10^4) for 10 min and 1h, and then fixed with paraformaldehyde. Cells were stained with Hoechst (10 nM; Invitrogen), images were captured using a Zeiss Axioplan microscope (Zeiss), and analyzed using Image J 1.46r software. Data shown as percentage of neutrophils with lobulated (control, ctr) and decondensed (netosis) nuclei after 10 min and 1h stimuli. At least 100 cells were analyzed in three different fields in three different donors. **$p<0.0001$ and *$p<0.05$.

Figure S6.

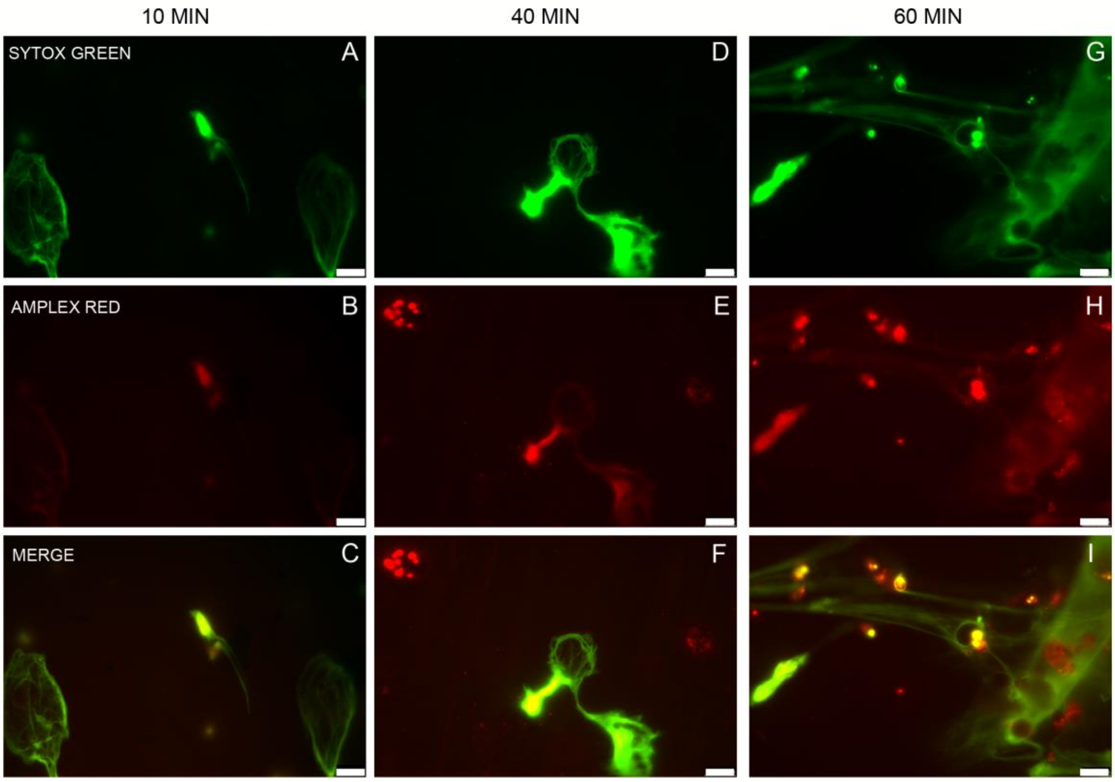

Figure S6. NET release induced by *Leishmania amazonensis* and peroxide production. Neutrophils and promastigotes were incubated with Amplex Red (5 µM) and Sytox green (0.5 µM). Cells were imaged alive at the indicated time points. NET release stained with Sytox green (A, D, G); Amplex red (B, E, H); (C, F, I) merged images. Bars: 10 µm.

Figure S7.

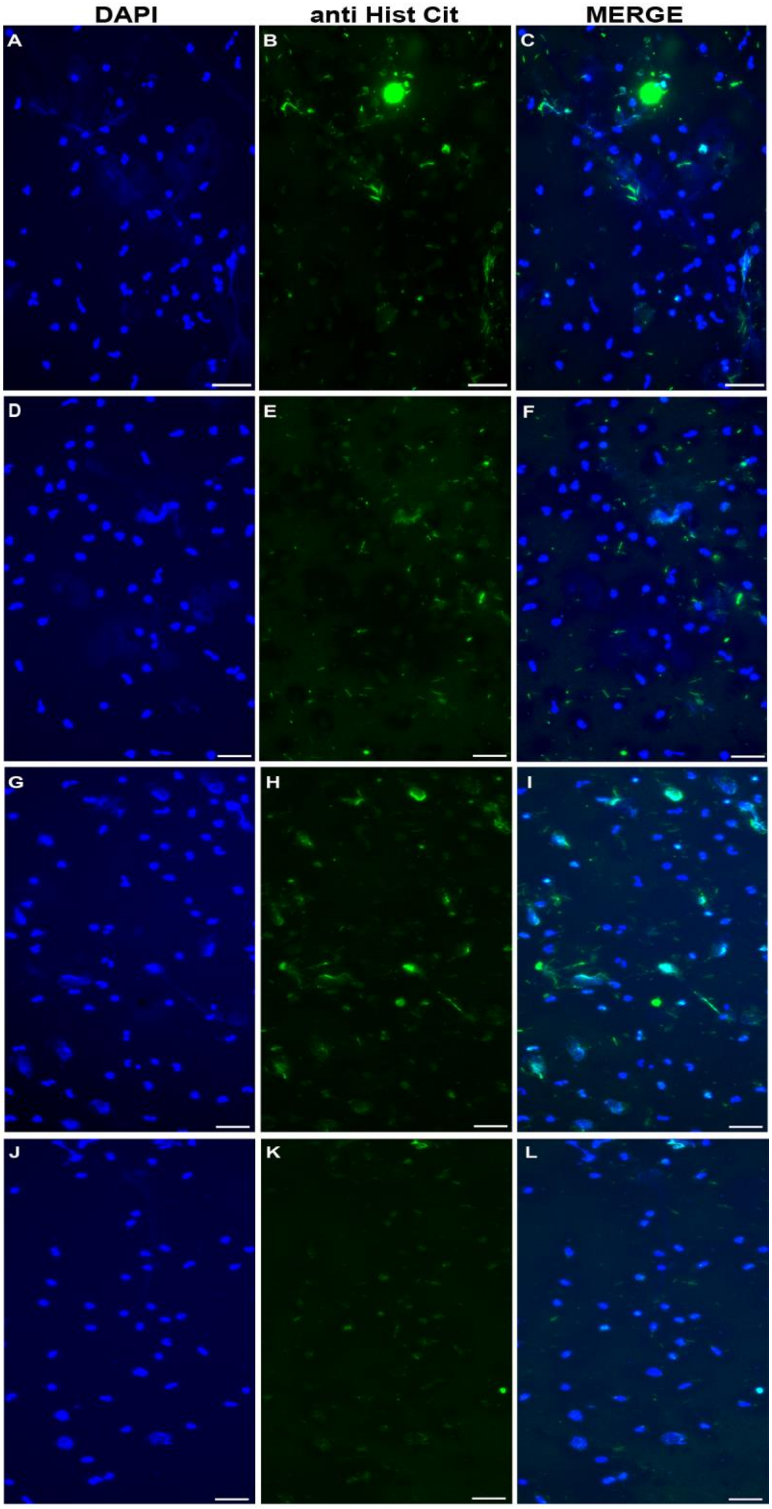

Figure S7. Immunostaining of Netosis induced by *Leishmania amazonensis*. Neutrophils untreated (A – F) or treated with clhoroamidine (G – L) were incubated with promastigotes for 10 min (A - C, and G - I) and 1 h (D - F, and J - L) at 35°C. Cells were fixed with paraformaldehyde and stained for NETs with DAPI (A, D, G, J; blue staining) and histone H3 citrullinated (B, E, H, K, green staining). Merged images (C – L). Bars: 30 µm.
